# The Effects of Tracking Responses and the Day of Mailing on Physician Survey Response Rate: Three Randomized Trials

**DOI:** 10.1371/journal.pone.0016942

**Published:** 2011-02-23

**Authors:** Elie A. Akl, Swarna Gaddam, Reem Mustafa, Mark C. Wilson, Andrew Symons, Ann Grifasi, Denise McGuigan, Holger J. Schünemann

**Affiliations:** 1 Department of Medicine, State University of New York at Buffalo, Buffalo, New York, United States of America; 2 Department of Family Medicine, State University of New York at Buffalo, Buffalo, New York, United States of America; 3 Department of Clinical Epidemiology and Biostatistics, McMaster University, Hamilton, Ontario, Canada; 4 Department of Internal Medicine, University of Iowa, Iowa City, Iowa, United States of America; Yale University School of Medicine, United States of America

## Abstract

**Background:**

The response rates to physician postal surveys remain modest. The primary objective of this study was to assess the effect of tracking responses on physician survey response rate (i.e., determining whether each potential participant has responded or not). A secondary objective was to assess the effects of day of mailing (Monday vs. Friday) on physician survey response rate.

**Methods:**

We conducted 3 randomized controlled trials. The first 2 trials had a 2×2 factorial design and tested the effect of day of mailing (Monday vs. Friday) and of tracking vs. no tracking responses. The third trial tested the effect of day of mailing (Monday vs. Friday). We meta-analyzed these 3 trials using a random effects model.

**Results:**

The total number of participants in the 3 trials was 1339. The response rate with tracked mailing was not statistically different from that with non-tracked mailing by the time of the first reminder (RR = 1.01 95% CI 0.84, 1.22; I^2^ = 0%). There was a trend towards lower response rate with tracked mailing by the time of the second reminder (RR = 0.91; 95% CI 0.78, 1.06; I^2^ = 0%). The response rate with mailing on Mondays was not statistically different from that with Friday mailing by the time of first reminder (RR = 1.01; 95% CI 0.87, 1.17; I^2^ = 0%), and by the time of the 2^nd^ reminder (RR = 1.08; 95% CI 0.84, 1.39; I^2^ = 77%).

**Conclusions:**

Tracking response may negatively affect physicians' response rate. The day of mailing does not appear to affect physicians' response rate.

## Introduction

Survey researchers employ various methods of questionnaire administration such as postal mail, electronic mail, online surveys, and phone interviews. Postal mail is commonly used as it is more convenient and efficient than phone surveys and provides higher response rates than electronic mail and online surveys [1]. Achieving high response rates is an important goal for generalizing results of surveys to the population it targets. However, the response rate to mail surveys has been trending down threatening the validity of the results [2]. Achieving adequate response rate is even more challenging with surveys of physicians [3].

Studies have shown that a number of strategies can improve the response rates to postal surveys [4]. These strategies include incentives [5], [6], [7], [8], [9], shorter length of the questionnaire [10], [11], [12], listing general questions first in the questionnaire [13], providing prompts to complete the survey [14], [15], and using certified (as opposed to first class) mailing [16]. However, all the above strategies appear to increase responses in small increments suggesting that combining a number of them may be necessary.

One factor that might affect the response rate is the day of the week on which the survey is received. This has been tested indirectly by comparing the day of the week on which the survey is mailed. Olivarius et al. conducted a survey on physicians, dispatching the questionnaire on Thursday versus Saturday and concluded that the probability of response was not influenced by receiving the postal questionnaire just before or just after the week-end [17]. Pressley et al found no statistical significant difference in response rates by mailing the surveys on Monday vs. Friday [18]. However his study was conducted on VIP executives, and not on physicians. Similarly, in a 2001 review of the literature, McColl et al concluded that the response rates are not affected by the day of mailing questionnaire [19].

Another factor that might affect response rate is tracking of responses, which refers to determining whether each potential participant has responded or not. Tracking is typically used to reduce time and cost expenditures of the survey by identifying and sending reminders to non-responders only. Tracking also helps in differentiating the demographic characteristics of responders from non-responders and in identifying any duplication of responses. Also, it allows record linkage when the same individuals are surveyed more than once over time. However, when the survey is on a sensitive topic, tracking responses might lower the response rates.

Asch surveyed nurses on topic of euthanasia and found that tracking responses (using coded post cards) lowered costs but significantly lowered the response rates [20]. Campbell et al [21] and McDaniel [22] surveyed the general public and did not find any significant difference in the response rates of anonymous and non-anonymous groups. McKee surveyed members of an organization and found that the response rate from the coded survey was substantially higher than that of the non-coded survey [23]. We identified no study conducted on physicians.

The primary objective of this study was to assess the effect of tracking responses on physician survey response rate. A secondary objective was to assess the effects of day of mailing (Monday vs. Friday) on physician survey response rate.

## Methods

We conducted 3 randomized controlled trials. The first 2 trials were conducted respectively among directors of Family Medicine and Internal Medicine residency programs in the United States (US). They used a factorial design to test the effect of day of mailing (Monday vs. Friday) and of tracking vs. no tracking responses. The third trial was conducted with a group of practicing physicians and tested the effect of day of mailing (Monday vs. Friday).

### Ethics statement

The University at Buffalo Institutional Review Board approved both studies. No informed consent was required and the University at Buffalo Institutional Review Board approved the waiver of consent.

### Two trials among program directors

#### Setting

We conducted the two trials in the setting of two national surveys of directors of Family Medicine and Internal Medicine residency programs in the US [24], [25]. The surveys related to training of residents in the implementation of clinical practice guidelines. The surveys' questionnaire consisted of 2 single sided pages with 15 questions about the curriculum, the characteristics of the program director and the characteristics of the residency program.

We mailed the initial invitation to participate in the surveys in April 2007. We used the following survey methods to maximize response rate [26], [27]: university sponsorship, personalized cover letter, colored ink, stamped return envelope, first class mailing, follow up mail, including a questionnaire in the follow up mail, non-monetary incentive, and a questionnaire that is interesting, short, user friendly, and with factual questions. The non-monetary incentive was mailed with the initial questionnaire and consisted of a Jeopardy-like game to teach clinical practice guidelines in a Microsoft PowerPoint file format on a CD.

#### Interventions

**Figure 1:**
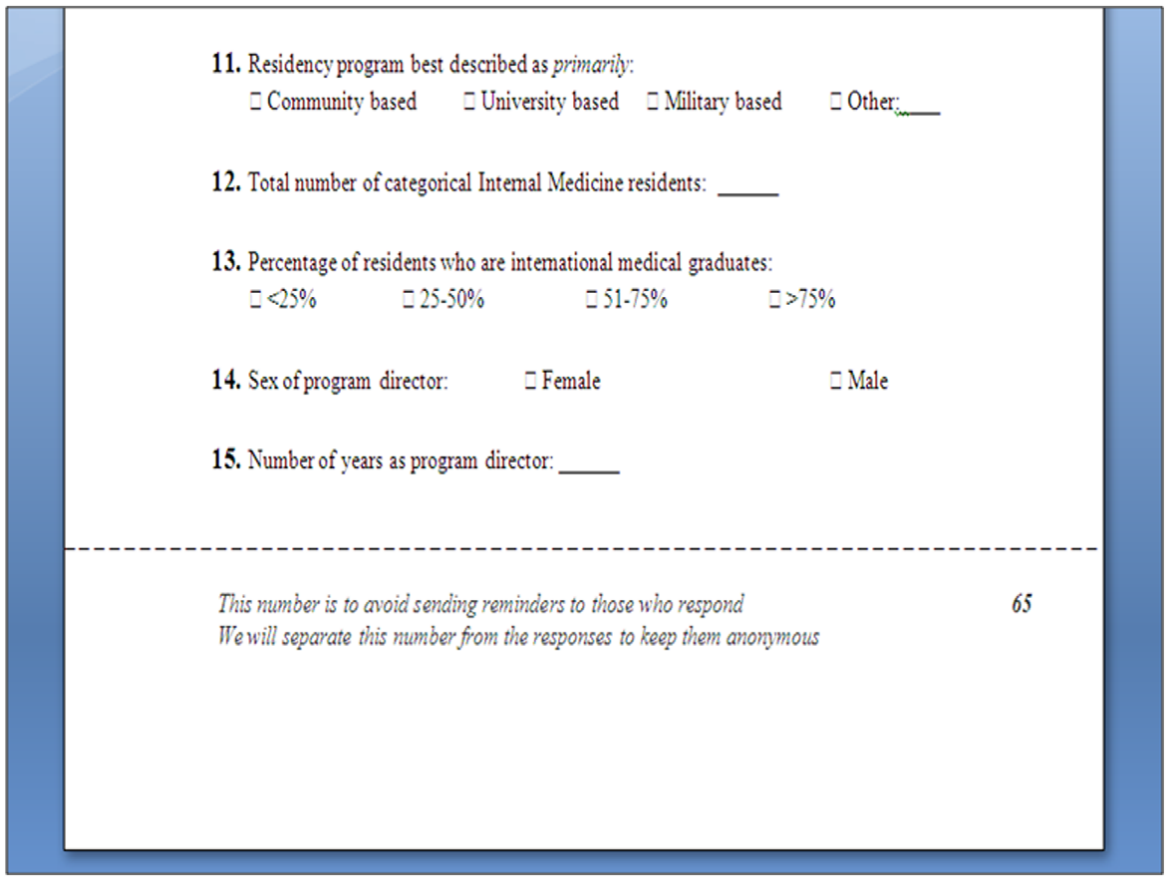
Image of a tracked questionnaire showing the perforation, the tracking number and a note explaining the purpose of tracking.

Each trial had a 2×2 factorial design to evaluate tracking responses and the day of mailing. We first randomized subjects to mailing the survey on a Friday versus a Monday. At the same time we randomized subjects to have their responses tracked versus not tracked. In the tracked response group, the second (and last) single sided page of the questionnaire was perforated at 3" from the bottom. Below the perforation were a tracking number and a message that read: “This number is to avoid sending reminders to those who respond. We will separate this number from the responses to keep them anonymous” (Figure 1). Upon the receipt of the response, we detached the lower portion of the page at the perforation line to keep the data anonymous. We used this method to identify responders, but also to reassure participants that we are ensuring anonymity.

Five weeks after the initial invitation, we sent a follow up mail to non-responders in the tracked response group and to all subjects in the non-tracked response group. Nine weeks after the initial invitation we sent faxes in attempt to increase response rate; we do not include data beyond the point of sending the faxes as we did not organize it according to the randomization scheme. We used Microsoft Excel to generate a list of random numbers for the allocation of subjects to one of the four study arms.

### Trial among practicing physicians

#### Setting

We surveyed Lebanese medical graduates practicing medicine in the United States regarding their intentions to relocate to Lebanon or Arabic Gulf countries (results of this survey not published yet). The questionnaire included 7 questions about family factors and 3 questions about willingness to relocate. We used the same techniques to improve response rate to this second trial as in the 1^st^ trial except for the use of an incentive.

#### Interventions

We conducted the trial to evaluate effect of the day of mailing on response rates. We randomized subjects to mailing the survey on a Friday versus a Monday. We sent the initial invitation to participate in the survey by first class postal mail. Two weeks later, we sent a first reminder by mail to non respondents. For those who still did not respond two weeks later, we sent a second reminder by fax when a fax number was available and attempted a phone contact as a reminder if a phone number was available.

### Statistical analysis

For the tracking comparison, we calculated for each of the 2 related trials (among program directors) the risk ratio for response rates by the time of first reminder and the risk ratio for the response rate by the second reminder. We then conducted a meta-analysis pooling the results of the 2 studies at each of the 2 times using a random effects model. This meta-analysis was not based on an exhaustive systematic review of the literature and thus did not include all potentially eligible studies. We tested results for homogeneity across studies using the I^2^ test. We followed a similar procedure for the day of the week comparison using the 3 related trials (among program directors and practicing physicians). We calculated the sample size to test the hypothesis of the survey [24], [25] and not specifically to test the hypothesis of the current trial. In other words, the primary outcomes based on which we conducted the power analysis related to the specific subject of the survey and not the surveying trial outcomes.

Concerns have been expressed about the potential for unrecognized interactions between interventions in factorial trials to distort their published results and interpretations [28]. We thus examined interaction between the 2 interventions in the factorial design trials (tracking and day of the week) by generating an “interaction ratio” that compares the effects of each treatment in the presence and absence of the other treatment [29]. The resulting interaction ratio and its 95% confidence interval showed no interaction [29].

## Results

The total number of participants in the 3 trials was 1339. Table 1 shows the number of participants randomized and the number responding for each trial and for each of the intervention arms. The response rates by the end of second reminder (not including fax and telephone reminders) for the 3 trials were 49% (Family Medicine), 39% (Internal Medicine) and 49% (practicing physicians' survey). The overall response rates by the end of the study for the 3 trials were respectively 52% (Family Medicine survey), 51% (Internal Medicine survey) and 57% (practicing physicians' survey).

**Table 1:** Number of participants randomized and number responding by the time of the 2^nd^ reminder for each trial and for each of the intervention arms.

		Intervention arm			
Trial		Non-tracked	Tracked	Monday	Friday
Family Medicine survey	# randomized	228	228	228	228
	# responding	115	106	93	128
	% responding	50.4	46.5	40.8	56.1
Internal Medicine survey	# randomized	191	192	192	191
	# responding	79	71	79	69
	% responding	41.4	37	41.1	36.1
Practicing physicians survey	# randomized	n/a	n/a	250	250
	# responding	n/a	n/a	122	125
	% responding	n/a	n/a	48.8	50

**Figure 2:**
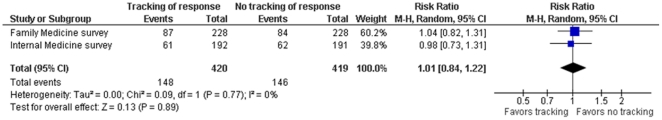
Meta-analysis of response by the time of first reminder comparing tracked vs. non-tracked mailing.

**Figure 3:**
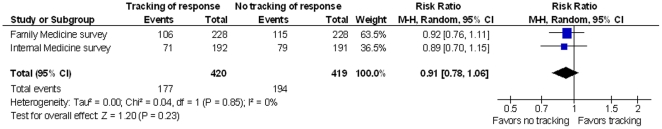
Meta-analysis of response by the time of second reminder comparing tracked vs. non-tracked mailing.

The risk ratios of response by the time of first reminder for tracked vs. non-tracked mailings for the Family Medicine and Internal Medicine surveys were 1.04 (95% CI 0.82, 1.31) and 0.98 (95% CI 0.73, 1.31) respectively. The pooled risk ratio of response by the time of first reminder was 1.01 (95% CI 0.84, 1.22; I^2^ = 0%) (Figure 2). The risk ratios of response by the time of second reminder for tracked vs. non-tracked mailings for the Family Medicine and Internal Medicine surveys were 0.92 (95% CI 0.76, 1.11) and 0.89 (95% CI 0.70, 1.15) respectively. The pooled risk ratio of response by the time of second reminder was 0.91 (95% CI 0.78, 1.06; I^2^ = 0%) (Figure 3).

**Figure 4:**
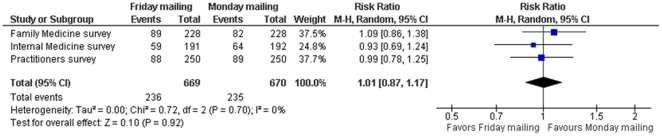
Meta-analysis of response by the time of first reminder comparing Friday vs. Monday mailing.

**Figure 5:**
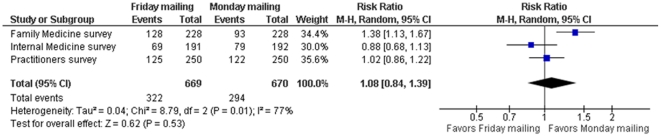
Meta-analysis of response by the time of second reminder comparing Friday vs. Monday mailing.

The risk ratios of response by the time of first reminder for Friday vs. Monday mailings for the Family Medicine, Internal Medicine and Practicing physicians' surveys were 1.09 (95% CI 0.86, 1.38), 0.93 (95% CI 0.69, 1.24), 0.99 (95% CI 0.78, 1.25). The pooled risk ratio of response by the time of first reminder was 1.01 (95% CI 0.87, 1.17; I^2^ = 0%) (Figure 4). The risk ratios of response by the time of second reminder for Friday vs. Monday mailings for the Family Medicine, Internal Medicine and Practicing physicians' surveys were 1.38 (95% CI 1.13, 1.67), 0.88 (95% CI 0.68, 1.13), 1.02 (95% CI 0.86, 1.22). The pooled risk ratio of response by the time of second reminder was 1.08 (95% CI 0.84, 1.39; I^2^ = 77%) (Figure 5).

## Discussion

We evaluated the effects of tracking responses and the day of mailing on physician survey response rate in three trials. The response rate with tracked mailing was not statistically different from that with non-tracked mailing by the time of the first reminder. There was a trend towards lower response rate with tracked mailing by the time of the second reminder. The response rate with Monday mailing was not statistically different from that with Friday mailing by the times of first reminder. The results corresponding to the time of the 2^nd^ reminder were inconsistent but suggest no effect.

Cost being one additional consideration with tracking mail, we conducted a post hoc cost analysis. The cost of follow-up mailing for the tracked group vs. non-tracked group was $455 and $649 respectively in the Internal Medicine survey, and $489 and $775 respectively in the Family Medicine survey.

This study has a number of strengths. We conducted three separate trials that we pooled together to improve the precision of our analyses and explore differences between the findings. We believe these are the first trials estimating the effects of tracking responses on response rates to surveys of physicians. We tested the interventions of interest while implementing most of the techniques shown to improve response rates. The heterogeneity across included trials was very low for 3 of 4 analyses which increase our confidence in the results.

One limitation of the study relates to the sample size that did not allow a more accurate estimation of effects despite the use of meta-analysis. Also, the response rates for these surveys were not high (the highest being 50.4 by the time of second reminder). One possibility, although unlikely, is that the increased response rates in the non-tracking group might be due to some respondents answering more than one questionnaire. Finally, the generalizability of these findings might be limited given the studies were conducted among physicians.

The trend toward a lower response rate with tracked mailing is consistent with the finding by Asch et al. that tracking using coded post cards lowered the response rates [20]. That survey was conducted on nurses was on sensitive topic of euthanasia. However, the results were not consistent with the remainder of the literature. Campbell and McDaniel conducted surveys on general population and customers of retail store respectively and did find any effect of anonymity on response rates. McKee et al. surveyed members of a national non-profit professional organization and used coded number at the top of front page of the questionnaire with explanation about the intent to follow-up. They found that coding actually increased the response rates. While these differing results might relate to the sensitivity of the topic, they might also depend on the type of population surveyed.

All the articles we identified on the effect of the day of mailing concluded that there is no statistically significant influence on the response rates [17], [18], [19]. In our study, we assumed that surveys mailed on Monday would improve response rate by reaching the participants by mid of the week when they might have more time to respond. Our findings do not support this assumption. In fact, the day of mailing may not be perfectly associated with the day of reading. The latter depends on when the mailroom at the receiving institution actually delivers the letter, when the residency director actually opens the letter, etc.

### Implications for conducting surveys

The decision to track responses will depend on the researchers' judgment whether the time and cost savings are worth the potential loss in response rate. One could assume that the less sensitive the survey topic is, the more the balance will be in favor of tracking. Also the survey could cost significantly less when the responses are tracked. As for the day of the week, we suggest that survey researchers not restrict their survey plans to a specific day of the week for the aim of improving response rate.

### Implications for research

Additional trials are needed to explore the interaction between the response rate with tracking responses and factors such as the sensitivity of the survey topic. Similarly, more trials are needed to explore the effect of mailing on different days of the week.
